# Detection of Innate Immune Response Modulating Impurities in Therapeutic Proteins

**DOI:** 10.1371/journal.pone.0125078

**Published:** 2015-04-22

**Authors:** Lydia Asrat Haile, Montserrat Puig, Logan Kelley-Baker, Daniela Verthelyi

**Affiliations:** Laboratory of Immunology, Division of Biotechnology Review and Research III, Office of Biotechnology Products, Center for Drug Evaluation and Research, Food and Drug Administration, Silver Spring, Maryland, United States of America; Virginia Tech University, UNITED STATES

## Abstract

Therapeutic proteins can contain multiple impurities, some of which are variants of the product, while others are derived from the cell substrate and the manufacturing process. Such impurities, even when present at trace levels, have the potential to activate innate immune cells in peripheral blood or embedded in tissues causing expression of cytokines and chemokines, increasing antigen uptake, facilitating processing and presentation by antigen presenting cells, and fostering product immunogenicity. Currently, while products are tested for host cell protein content, assays to control innate immune response modulating impurities (IIRMIs) in products are focused mainly on endotoxin and nucleic acids, however, depending on the cell substrate and the manufacturing process, numerous other IIRMI could be present. In these studies we assess two approaches that allow for the detection of a broader subset of IIRMIs. In the first, we use commercial cell lines transfected with Toll like receptors (TLR) to detect receptor-specific agonists. This method is sensitive to trace levels of IIRMI and provides information of the type of IIRMIs present but is limited by the availability of stably transfected cell lines and requires pre-existing knowledge of the IIRMIs likely to be present in the product. Alternatively, the use of a combination of macrophage cell lines of human and mouse origin allows for the detection of a broader spectrum of impurities, but does not identify the source of the activation. Importantly, for either system the lower limit of detection (LLOD) of impurities was similar to that of PBMC and it was not modified by the therapeutic protein tested, even in settings where the product had inherent immune modulatory properties. Together these data indicate that a cell-based assay approach could be used to screen products for the presence of IIRMIs and inform immunogenicity risk assessments, particularly in the context of comparability exercises.

## Introduction

Immune responses to protein therapeutic products, even those with high homology to human sequences are frequent and can significantly affect the safety and efficacy of therapeutic proteins and peptides [1–4]. Thus assessing the risk of a therapeutic product inducing an immune response prior to its clinical use is important and requires a thorough understanding of the product, including its structure, manufacturing process, mechanism of action and bio-distribution. Biologics, whether recombinant or naturally derived, are manufactured using complex expression/production systems that usually involve a genetically modified host cell (bacteria, yeast, plant, insect or mammal) and growth/fermentation media. While the downstream purification processes are designed to eliminate most impurities, the level and types of product and process related impurities in the final drug is dependent on the purification process and could be modified by manufacturing changes [5]. These impurities could include host cell proteins and microbial structures, as well as other organic or inorganic components. In a recent study we showed that some of these impurities can act directly on receptors of the innate immune system and facilitate the development of an immune response [5].

The innate immune system is armed with a variety of gene-encoded pattern recognition receptors (PRR) that recognize and get activated by pathogen associated molecular patterns (PAMPs). Each of these receptors is activated by unique microbial structures [6,7] that evoke responses that are primarily channeled through the activation of NF-κB and AP1, resulting in the production of pro-inflammatory cytokines (IL-6, TNF-α, IFNs), reactive oxygen species (ROS) and chemokines (CXCL8/IL-8, CCL5, CXCL10) as well as increased antigen uptake, processing and presentation by antigen presenting cells. If these are delivered together with a therapeutic protein, they may attract and activate immune cells to the site of the product facilitating the generation of an immune response [8]. The best characterized families of receptors that recognize IIRMIs are the Toll-like receptors (TLR). In human cells, TLR4 can be activated by endotoxin present in gram negative bacteria, β-glucans from yeast as well as heat shock proteins or heparin sulfate fragments [9–10]. Similarly, TLR2 mediates response to lipoproteins, glycolipids, lipoteichoic acids and zymosan. Ligands for other receptors appear to be more restricted, for example TLR5 responds to flagellin, TLR3 and TLR7 respond to ds and ssRNA respectively, and TLR9 is known to be activated by specific DNA motifs as well as hemozoin from malaria parasites [11–12]. Most TLR ligands are known to act as adjuvants increasing antigen uptake and presentation, T cell activation and antibody production. Importantly, there is ample evidence supporting the existence of other PAMPS binding c-type lectin receptors (CLRs), Nod-like receptors (NLRs) and RLG-I like receptors (RLRs) with similar adjuvant effect [13]. Furthermore, new receptors continue to be identified that can trigger an immune response such as environment pollutant sensor aryhydrocarbon receptor (AhR) and caspase 11, which are activated by bacterial pigments and intracellular LPS respectively [14–15]. Previous studies have shown that trace levels of PRR ligands can act alone or synergize to increase the immunogenicity of a co-delivered therapeutic proteins such as erythropoietin [5].

Despite mounting evidence showing that low levels of process and product related impurities can act as PAMPs triggering an innate immune response, currently only a fraction of the potential IIRMIs (endotoxin and DNA) are routinely measured in drug substance or drug product. This is partly due to the lack of sensitive and reliable screening tests [16]. The development of methods to characterize IIRMIs has been hampered by several factors including the breadth and complexity of potential impurities. This complexity dictates that a suitable method to inform the risk of product immunogenicity would need to be sensitive to known and unknown impurities present at trace levels alone or as mixtures of several IIRMIs in therapeutic protein products. Our studies describe two possible approaches to detect the presence of IIRMIs that might inform the risk assessment for immunogenicity of a therapeutic protein.

## Materials and Methods

### Reagents

LPS from *Salmonella Minnesota* Re 595 was purchased from Calbiochem (Darmstadt, Germany). Pam3CSK4, Poly I:C, flagellin, FSL-1, imiquimod, CLO75, MDP and zymosan were purchased from InvivoGen (San Diego, CA, USA). CpG ODN 1555 (GCTAGACGTTAGCGT) and CpG ODN B2006 (TCGTCGTTTTGTCGTTTTGCTGTT) were synthesized at the FDA core facility (Rockville, MD, USA) and used at the concentration indicated in each individual figure. The purity of each TLR agonists was established using a panel of HEK-BLUE cell lines transfected with individual TLRs (HEK-BLUE-hTLR2, 4, 5, 7 and 9) from InvivoGen (San Diego CA, USA). Commercial lots of Erythropoietin (Procrit) IFNβ-1a (Avonex), and heparin were used.

### Cell culture

#### RAW-BLUE

Murine Raw 264.7 macrophages carrying a SEAP reporter construct inducible by NF-κB were purchased from InvivoGen. Cells were grown in DMEM supplemented with 10% FCS, 2mM L-glutamine, 100μg/mL Normocin in the presence of selection antibiotic 200μg/mL Zeocin and passaged when 70% confluence was reached per manufacturer’s recommendation. Cells were scraped and resuspended in RAW-BLUE test media (DMEM, 10% heat inactivated FBS, 100μg/mL Normocin and 2mM L-glutamine) for testing.

#### MM6

Human monocytic cell line Macrophage-like-MonoMac6 (MM6) cells (Germany Culture Collection) [17], were a generous gift from Marina Zaitseva (CBER, FDA), were grown in RPMI 1640 containing 15% FBS, 2mM Glutamate, 1mM non-essential amino acid and 1mM Sodium Pyruvate and 100IU/mL Pencillin-streptomycin, 10μg/ml Insulin-Transferrin-Selenium (Life Technologies, Carlsbad, CA) with 1mM oxaloacetic acid purchased from Sigma-Aldrich (St. Louis, MO). Cell passage was limited to 25.

#### THP-1-TNF-α luciferase reporter cells

THP-1 cells with a TNF-α promoter driven luciferase reporter were obtained from Iain Fraser (NIAID, NIH) [18]. THP1-TNF-α luciferase reporter cells were maintained in RPMI-1640 media supplemented with 10% FBS, 10mM HEPES, β-mercaptoethanol, 2mM L-glutamine and 500ug/mL geneticin sulfate (Life technologies). Cells were plated at 200,000/mL and differentiated with 5ng/mL PMA for 72h. Following differentiation, the cells were stimulated for 4h with increasing concentration of TLR ligands as indicated on the figures. Luciferase activity was measured using Bright Glo-luciferase (Promega, Madison, USA) assay system per manufacturer’s instructions.

#### HEK-BLUE hTLR transfectants

HEK-BLUE-hTLR2 and HEK-BLUE-hTLR4 cells were cultured in DMEM 10% FCS with 50μg/mL penicillin-streptomycin, 100μg/mL normocin, 2mM L-glutamine supplemented with 1X HEK-BLUE Selection. Whereas HEK-BLUE-hTLR5, HEK-BLUE-hTLR7 and HEK-BLUE-hTLR9 cells were maintained with growth media supplemented with Blasticidin and Zeocin as per manufacturer instructions.

### Stimulation of NF-κB reporter cells with TLR ligands

Cells were plated at the density indicated on each figure legend in flat bottom 96-well plates in a 100μL final volume and a serial dilution of respective TLR ligands were prepared and added at 100μL volume as indicated in figure. After 24h of stimulation, supernatants were collected and NF-κB activation was determined using detection medium QUANTI-Blue prepared according to manufacturer recommendations. Briefly, 150μL of QUANTI-Blue were added to 96-well flat bottom plate together with 50μL of supernatant from the TLR induced samples. After 2h of incubation at 37°C, the SEAP levels were determined colorimetrically at 620 nm by spectrophotometry.

### PBMC stimulation

Deidentified buffy coats were obtained from the NIH blood bank, (Bethesda, MD, USA). PBMC were isolated by density-gradient centrifugation over Ficoll-Hypaque. PMBC were cultured at 37°C in complete RPMI medium in 96-well plates at a density of 2.5x10^6^ cells/mL in quadruplicate. Individual TLR ligands were added at the beginning of the culture as stated in the figure legend. After 24h of stimulation, PBMC were collected, lyzed with TRIzol reagent (Invitrogen, Carlsbad, CA), and stored at -80°C until further analysis.

### qRT-PCR analysis

Cytokine mRNA measurement following stimulation of MM6 or PBMC as well as TLR mRNA expression was performed by qRT-PCR. Total RNA was prepared from cells lysate using TRIzol (Invitrogen, Carlsbad, CA) as per manufacturer instructions. Contaminating genomic DNA was removed with TurboDNase (Ambion, Austin, TX). Subsequently RNA (1μg/mL) was reverse transcribed into cDNA using high capacity cDNA Reverse Transcription Kit (Applied Biosystem, Foster City, CA) as per manufacturer recommendation. The PCR reactions were conducted in 1x Universal master mix (Applied Biosystem, Foster city, CA) with 1/20 volume cDNA/reaction for individual gene expression assays, in a Viia7 Real-time PCR system (Applied Biosystem, Foster city, CA). Change in the expression levels of IL-6 and IL-8 mRNA in stimulated cultures were indicated as fold increase over unstimulated cells using 2^-ΔΔCt^ unless otherwise mentioned in the figure legend. TLR expression is normalized to 18S and shown as ΔCt.

### Statistical analysis

Individual data points represent the mean ± SD of 3 biological replicates repeated in at least 2 separate experiments. Statistical comparisons were assessed by Student’s *t*-test or One-way ANOVA followed by Tukey test as appropriate. Statistical analyses were performed with Graph pad Statistical Software (Graph Pad Software Inc., San Diego, CA, USA). Statistical significance was defined as *P*<0.05.

## Results

### Utilization of hTLR expressing cells lines to detect IIRMIs

**Fig 1:**
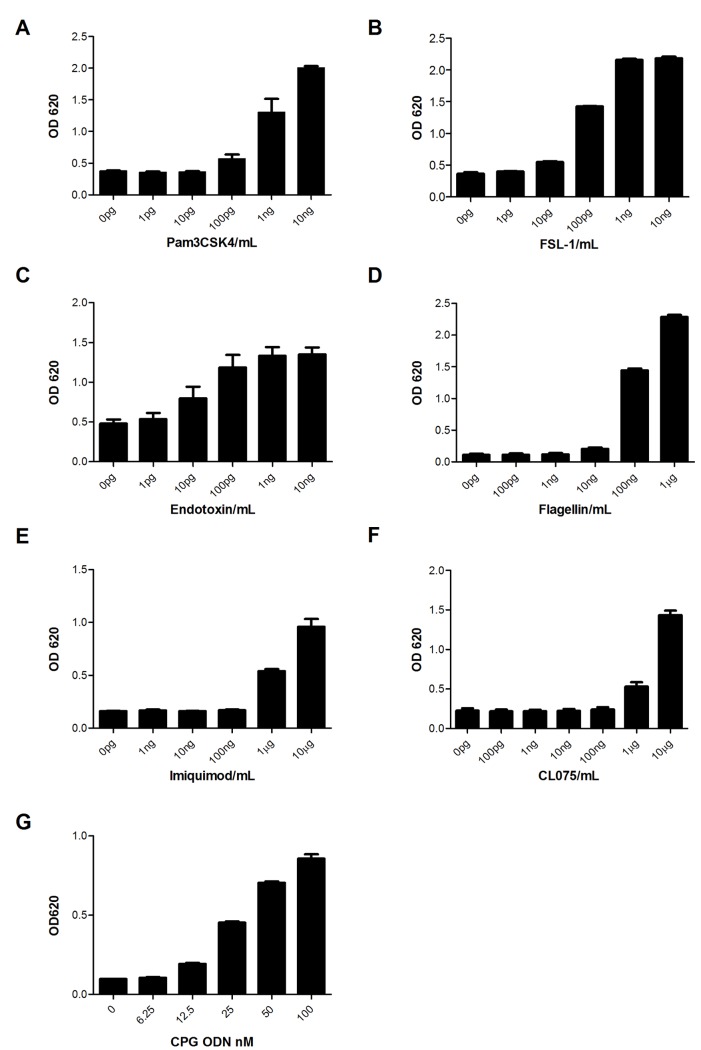
Dose response analysis of HEK-BLUE-hTLR transfectants to TLR stimulating IIRMIs. **(A/B)** A 200uL of cell suspension containing 5x10^4^ HEK-BLUE-hTLR2 **(C)** 2.5x10^4^ HEK-BLUE-hTLR4 **(D)** 2.5x10^4^ HEK-BLUE-hTLR5 **(E & F)** 4x10^4^ HEK-BLUE-hTLR7 **(G)** 8x10^4^ HEK-BLUE-hTLR9 cells were cultured with increasing level of their respective ligands Pam3CSK4, FSL-1, Endotoxin, Flagellin, Imiquimod/CLO75 and CpG ODN B2006 for 24h. The level of NF-κB activation in the supernatant was measured using QUATI-BLUE as described in materials and methods. Each point represents mean ± SD of from 2 independent experiments.

Since PRR are highly expressed in PBMC, a mixture of human immune cells such those found in PBMC should provide a comprehensive detection system for IIRMIs that is relevant to the clinical scenario. However, efforts to use PBMC as reporter systems have been thwarted by donor to donor variability, the complexity of obtaining, preparing and storing human PBMC and the inherent difficulties in validating assays based on primary cells [19–20]. HEK-293 cells stably transfected with different human TLRs and an NF-κB-inducible reporter gene (HEK-BLUE-hTLR) offer an interesting alternative as they are commercially available and their use would be easier to validate. Therefore, we explored the capability of these cell lines to detect low levels of IIRMIs as well as their suitability to detect low levels of impurities in therapeutic proteins. First we tested the sensitivity of the cell lines to purified TLR ligands specific for the individually expressed TLR. HEK-BLUE-hTLR2 were activated by concentrations of Pam3CSK4 (Fig 1A) or FSL-1 as low as 100pg/mL and 10pg/mL respectively (Fig 1B). Likewise, activation of NF-κB in HEK-BLUE-hTLR4 was achieved at a concentration as low as 10pg/mL of endotoxin (Fig 1C). Similarly, HEK-BLUE-hTLR5 cells were stimulated by 100ng/mL of flagellin (Fig 1D) and HEK-BLUE-hTLR7 cells required 1μg/mL of Imiquimod or CLO75 to be activated (Fig 1E and 1F). The lowest concentration of CpG ODN to consistently induce NF-κB activation in HEK-BLUE-hTLR9 was 12.5nM (100ng/mL) (Fig 1G), similar to what was previously published in mouse splenocytes [5]. The response observed in this panel of TLR expressing HEK-BLUE cells to increasing concentration of IIRMIs showed that the sensitivity of TLR transfected cells is comparable to that of human PBMC (Table 1). Importantly these cell lines were highly specific for the targeted impurity (Table 1). This was confirmed by stimulating each cell line with its own TLR ligand as well as other IIRMIs. Of note, all the transfected cell lines were activated by high levels of flagellin suggesting that the parental cell line has an intrinsic receptor for flagellin (Table 1). This is in agreement with the study by Tallant *et al*, which showed that HEK293 abundantly express TLR5 but weakly respond to flagellin, due to the lack of co-receptor or adaptor molecule required for efficient propagation of signal [21].

**Table 1:** Specificity of hTLR transfected HEK-BLUE cells towards their own ligand.

TLR ligand	HEK-BLUE wt	HEKBLUE hTLR2	HEKBLUE hTLR4	HEK-BLUE hTLR5	HEK-BLUE hTLR7	HEK-BLUE hTLR9	PBMC
**Pam3CSK4**	<LLOD	***100pg/mL***	<LLOD	<LLOD	<LLOD	<LLOD	1ng/mL
**Endotoxin**	<LLOD	<LLOD	***10pg/mL***	<LLOD	<LLOD	<LLOD	100ng/mL
**FSL-1**	<LLOD	***10pg/mL***	<LLOD	<LLOD	<LLOD	<LLOD	1pg/mL
**Flagellin**	10μg/mL	10μg/mL	10μg/mL	***100ng/mL***	10μg/mL	10μg/mL	5μg/mL
**Imiquimod**	<LLOD	<LLOD	<LLOD	<LLOD	***1μg/mL***	<LLOD	<LLOD
**CLO75**	<LLOD	<LLOD	<LLOD	<LLOD	***1μg/mL***	<LLOD	100ng/mL
**CpG**	<LLOD	<LLOD	<LLOD	<LLOD	<LLOD	***100ng/mL***	100ng/mL

**Fig 2:**
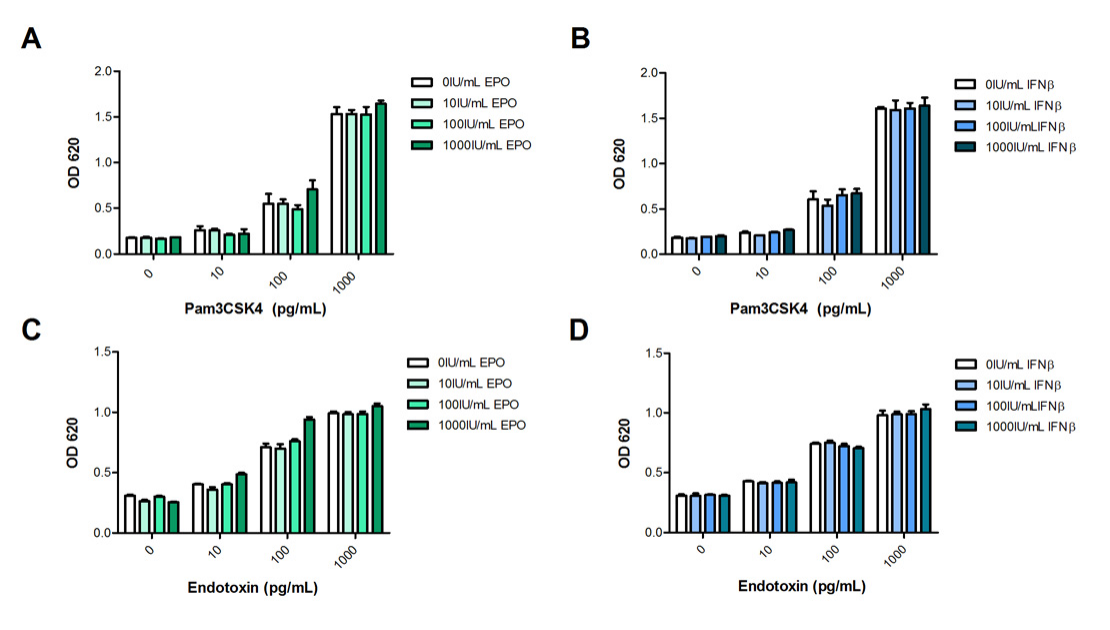
HEK-BLUE-hTLR transfectants detect trace levels of impurities in therapeutic products. (**A-B**) A total of 5x10^4^ HEK-BLUE-hTLR2 (**C-D)** 2.5x10^4^ HEK-BLUE-hTLR4 were cultured for 24h with increasing concentrations of Pam3CSK4 and Endotoxin in the presence of 0-1000IU/mL of EPO and IFNβ to assess the sensitivity of the cell lines and the product interference. NF-κB activation was measured from the supernatant after the addition of QUANTI-Blue. Each point represents mean ± SD of triplicate cell culture.

While the reporter cell lines are sensitive to purified IIRMIs, therapeutic biologics could mask or interfere with the response of the cells lines to IIRMIs therefore we next confirmed the sensitivity of each cell line to its corresponding TLR ligand in the presence of selected therapeutic proteins. As shown in Fig 2, HEK-BLUE-hTLR2 cells were cultured in the presence of increasing concentrations of ligand alone or together with increasing concentrations of recombinant human erythropoietin (EPO) or recombinant human interferon β (IFNβ). While the addition of EPO or IFNβ alone, even at a 1000IU/mL, did not impact the basal NF-κB activation on HEK-BLUE-hTLR2 cells, the addition of 100pg/mL of Pam3CSK4 to the product resulted in consistent NF-κB activation. The response of the cell line was dose dependent and unchanged by the presence of the therapeutic protein (Fig 2A and 2B). Similar results were observed when EPO or IFNβ were tested in HEK-BLUE-hTLR4 cells, in which case the lower limit of detection (LLOD) for endotoxin remained at 10pg/mL irrespective of inclusion of products to the cell culture system (Fig 2C and 2D). Together these data indicate that individual hTLR-transfected cell lines may be a useful tool to identify several IIRMIs, and the sensitivity of the HEK-BLUE-hTLR transfected cell lines to detect IIRMIs is not changed when the IIRMIs were co-administered with the product, however, the range of IIRMIs that can be tested in this system is restricted by the availability of stably transfected cell lines. In addition, caution should be taken that some of the cells may respond to more than the specified stimuli.

### Use of RAW-BLUE, THP-1 and MM6 cells to screen products for IIRMI

**Fig 3:**
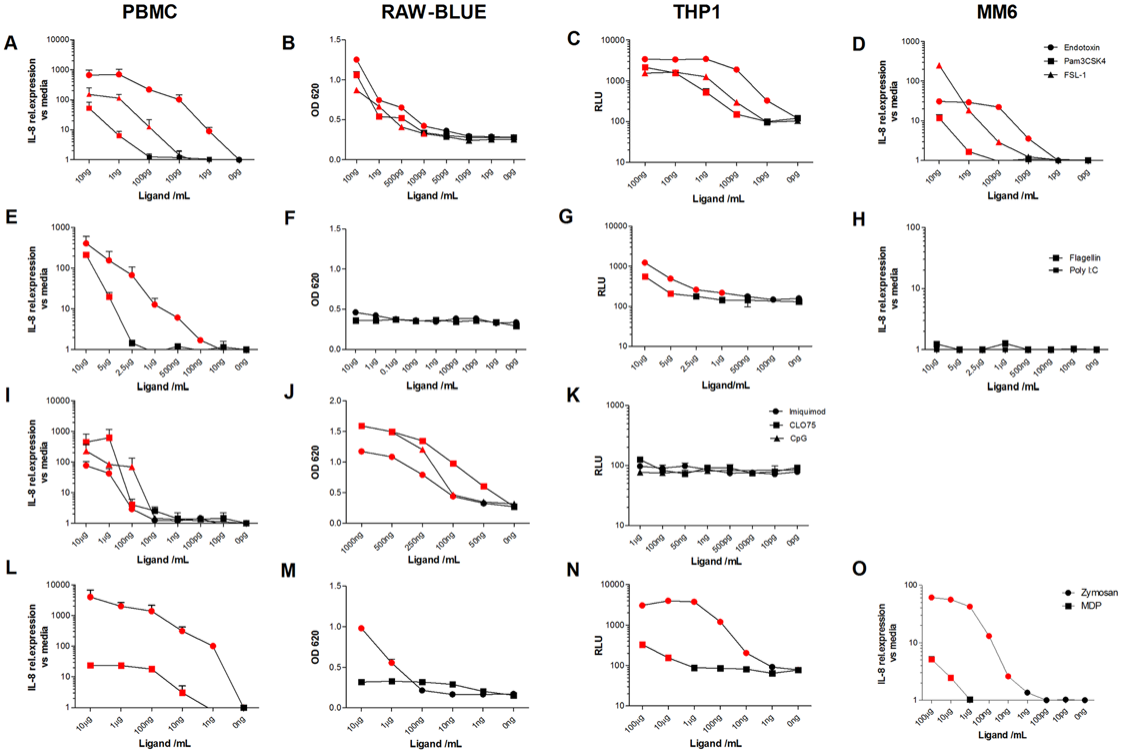
Limit of detection to PRR ligands in PBMC and monocyte/macrophage cell lines. PBMC, RAW-BLUE, THP-1 and MM6 cells were stimulated with the indicated concentration of (**A-D**) Endotoxin, Pam3CSK4 and FSL-1, (**E-H**) Poly I:C and Flagellin, (**I-K**) Imiquimod, CLO75 and CpG (**L-O**) zymosan and MDP. Levels of IL-8 mRNA (relative expression vs media), NF-κB activation (OD 620) and luciferase activity (RLU) were measured as described in materials and methods. All samples were tested in triplicate. Data points represent mean ± SD. (*p<0.05 vs media control). Red points show the limit of detection for each individual IIRMIs.

Given that product and process-related impurities are likely to be complex and stimulate the innate immune system through several receptors some of which may not have been identified as yet [22], we reasoned that screening cell lines that bear multiple receptors may provide a better platform. Thus we next determined whether a monocyte or macrophage cell line could be used to screen products for IIRMIs in the absence of prior knowledge of the complete set of impurities likely to be in a product [23–24]. To model this principle we selected 3 reporter cell lines, each with different readout systems: RAW-BLUE (SEAP reporter construct inducible by NF-κB), THP-1 (TNF-α promoter driven luciferase reporter) and MM6 (levels of mRNA IL-6 and IL-8 transcripts in response to PRR activation). Assessment of their PRR gene expression showed that none of the cell lines tested expressed a complete receptor repertoire but, when considered as a test panel they offered a more complete PRR spectrum to detect broader array of potential innate IIRMIs (S1 Fig). The expression of TLR1, TLR2, TLR4 and TLR6 was higher than those of TLR3 or TLR5 for the three cell lines tested. On the other hand, MM6 and THP1 cells expressed very low levels of mRNA for TLR7, TLR8 and TLR9 as compared to RAW-BLUE cells. The sensitivity of each cell line was compared to human PBMCs for individual ligands by placing them in culture with decreasing concentrations of: Pam3CSK4 (TLR1/2 ligand), Poly I:C (TLR3 ligand), endotoxin (TLR4 ligand), flagellin (TLR5 ligand), FSL-1 (TLR 2/6 ligand), imiquimod (TLR7 ligand), CLO75 (TLR 7/8 ligand), CpG ODN (TLR9 ligand), zymosan (TLR2/ Dectin 1 ligand), and muramyl dipeptide (MDP-NOD2 ligand). As shown in Fig 3, the LLOD of each ligand differs for each cell line. For example, human lines THP-1 and MM6 were activated by 10pg/ml of endotoxin while mouse line RAW-BLUE required 100pg/ml. Similarly, THP-1 cells were more sensitive to the presence of Pam3CSK4 (100pg/ml) than RAW-BLUE or MM6 cells (500pg/ml). THP-1 cells detected Poly I:C and flagellin at concentrations of 1μg/mL and 5μg/mL respectively, whereas RAW cells or MM6 did not. In contrast, only the RAW-BLUE cells were able detect nucleic acid ligands signaling through endosomal receptors TLR7, 8 and 9, although not by TLR3 and MM6 recognized MDP while RAW-BLUE and THP-1 cells did not. Lastly, zymosan, which requires a heterodimer of Dectin 1/TLR2 was detected by all cell lines albeit at relatively high concentrations (>10ng/mL) (Table 2). Interestingly, when combined, the three monocyte/macrophage cell lines provided similar sensitivity as PBMC for the detection of most ligands except for Poly I:C or MDP (Table 2). This suggests that the use of alternative cell lines may need to be considered to test products where the presence of TLR3 or NOD2 ligands is a concern. Overall these studies show that while none of the individual monocyte/macrophage cell lines could be expected to detect a complete spectrum of IIRMIs that could be present in the product, the combined use of several cell lines broadened the overall coverage and sensitivity of the system to resemble that offered by PBMCs.

**Table 2:** Limit of detection for PPR ligands by monocyte/macrophage cell lines and PBMC.

TLR ligand	RAW-BLUE	MM6	THP1	PBMC
**Pam3CSK4**	500pg/mL	500pg/mL	100pg/mL	1ng/mL
**Poly I:C**	<LLOD	<LLOD	1μg/mL	100ng/mL
**Endotoxin**	100pg/mL	10pg/mL	10pg/mL	1pg/mL
**Flagellin**	<LLOD	<LLOD	5μg/mL	5μg/mL
**FSL-1**	100pg/mL	100pg/mL	100pg/mL	100pg/mL
**Imiquimod**	100ng/mL	<LLOD	<LLOD	100ng/mL
**CLO75**	50ng/mL	<LLOD	<LLOD	100ng/mL
**CpG**	60ng/mL	<LLOD	<LlOD	100ng/mL
**Zymosan**	1μg/mL	10ng/mL	10ng/mL	1ng/mL
**MDP**	<LLOD	10μg/mL	<LLOD	10ng/ mL

### Effect of therapeutic proteins on the detection of IIRMIs by macrophage/monocyte cell lines

**Fig 4:**
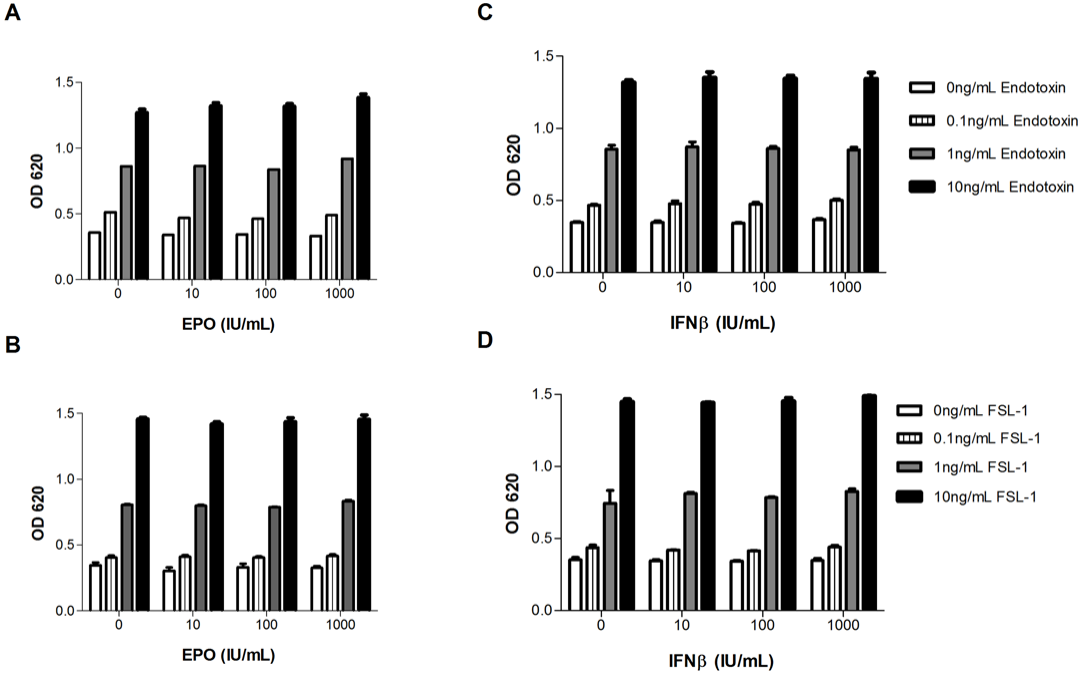
Effect of therapeutic protein concentration on screening assay. Titration of endotoxin and FSL-1 were performed in the presence of increasing concentration of EPO (**A-B**) and IFN-β (**C-D**) using RAW-BLUE cell. Supernatant was collected after 24h and NF-κB activation was measured by the addition of QUATI-BLUE medium and OD was read at 620. All samples were in triplicate. Data points represent mean ± SD.

**Fig 5:**
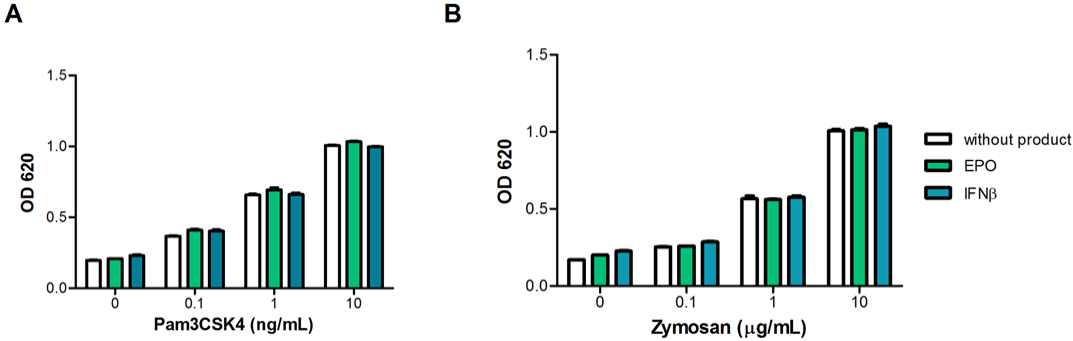
Therapeutic proteins don’t modify the LLOD in RAW—BLUE cells. RAW-BLUE cells were stimulated with indicated concentration of (**A**) Pam3CSK4 (**B**) Zymosan in the presence or absence of erythropoietin (1000IU/mL) and IFNβ (1000IU/mL) for 24h. NF-κB activation was assessed in the culture supernatant as described in materials and methods. Each point represents mean ± SD of triplicate cell culture.

We next examined whether the sensitivity to IIRMIs in these cell lines would be maintained in the presence of therapeutic products. Hence, the sensitivity of RAW-BLUE cells to endotoxin and FSL-1 was tested using a range of concentration of therapeutic proteins EPO and IFNβ. These cells do not express receptors for either one of the proteins. As shown in Fig 4, the addition of increasing concentration of EPO and IFNβ (10 IU/mL to 1000IU/mL) did not alter the sensitivity or the linearity of RAW-BLUE cells for the added IIRMIs, within the range of concentrations tested (and indicated in the corresponding figure). As shown in Fig 5, similar results were observed when Pam3CSK4 and zymosan were used to model IIRMI suggesting that RAW-BLUE cells can be used to screen IIRMIs in therapeutic proteins. Lastly, a consistent increase in TNF-α was evident when RAW-BLUE cells were used to test different lots of heparin spiked with 100pg of endotoxin (S2 Fig).

**Fig 6:**
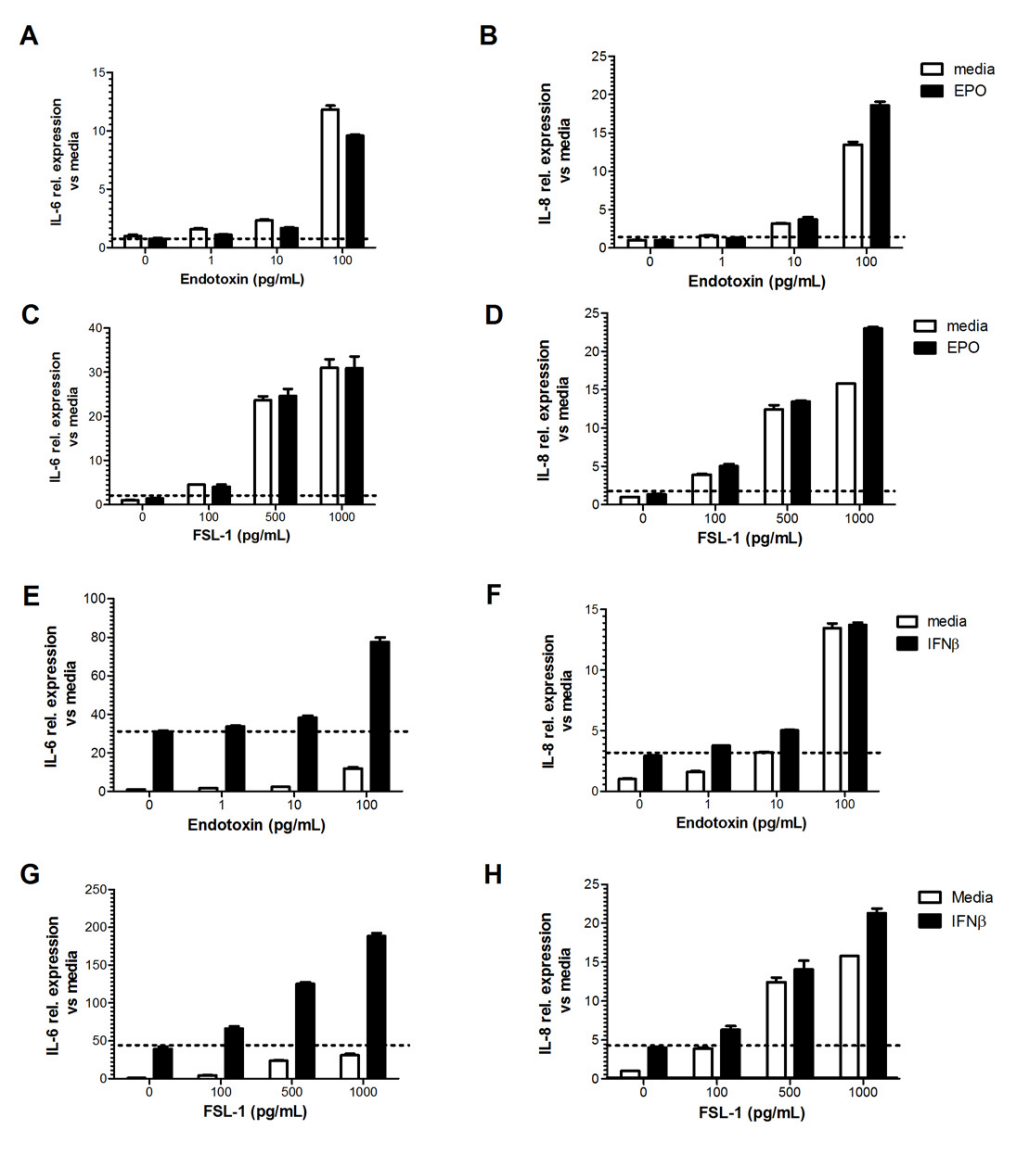
Impact of immunomodulatory proteins on the LLOD for PRR ligands in MM6 cells. MM6 cells were placed in culture with EPO (1000IU/mL) together with increasing concentration of (**A-B**) Endotoxin and (**C-D**) FSL for 24h. Messenger RNA expression of IL-6 and IL-8 was determined by q-RT-PCR. MM6 cells were placed in culture with IFNβ (1000IU/mL) together with increasing concentration of (**E-F**) Endotoxin and (**G-H**) FSL for 24h. Messenger RNA expression of IL-6 and IL-8 was determined by q-RT-PCR. A representative of 2 independent experiments with similar results is shown. (*p<0.05 vs EPO alone or IFNβ alone).

Next we examined if macrophage cells lines can be used to screen for IIRMIs in scenarios where the product *per se* would modulate the activity of the reporter cell line. To test this, human EPO or IFNβ were added alone or together with endotoxin and FSL-1 on human macrophage cell line (MM6), which bares receptors for IFNβ but not erythropoietin. As with the RAW-BLUE cells, addition of EPO did not impact the threshold of detection or linearity of response of MM6 cells to either endotoxin or FSL-1, however IFNβ induced the expression of IL-6 and IL-8 by MM6 cells in the absence of added IIRMIs. Importantly, despite the inherent stimulatory effect of the product, the addition of trace levels of endotoxin or FSL-1 to the cultures resulted in significant increase in the expression of IL-6 and IL-8 (Fig 6E–6H). In the case of IFNβ, the absence of a response using the murine RAW-BLUE cell line provided additional assurance that the commercial product did not contain detectable levels of IIRMIs, however, for products the use of this type of screening system may be restricted to monitoring changes in the response as part of comparability exercises. Together these data suggest that for therapeutic proteins that could modulate the activity of the screening cell line, a thorough understanding of the biology of the product in selecting the screening cell lines for detection of IIRMI, as well as additional controls may be needed to differentiate between the response of the reporter cells to the product and the response to potential impurities in the product.

### Detection of complex IIRMIs in therapeutic proteins

**Fig 7:**
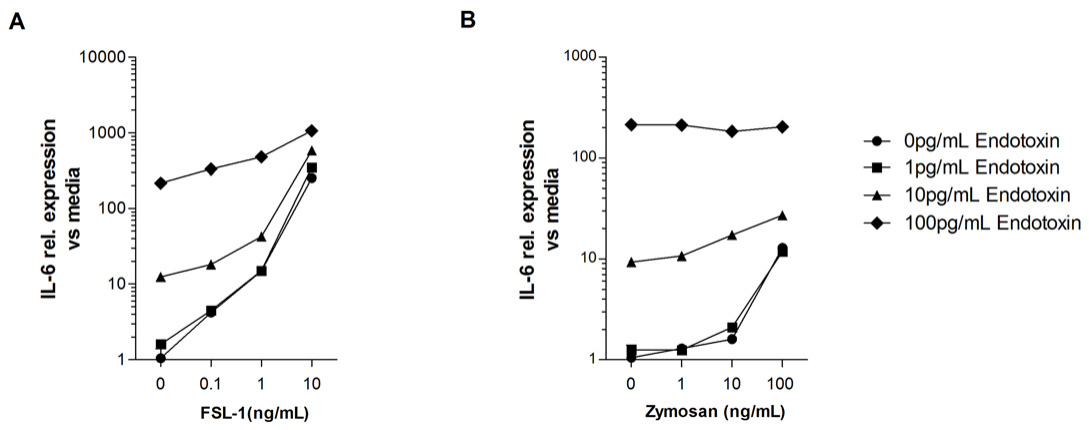
Multiple impurities do not modify the LLOD for each individual impurity. MM6 cells were cultured in the presence of increasing concentration of (**A**) Endotoxin and FSL-1 (**B**) Endotoxin and zymosan alone or in combination. mRNA level of IL-6 was quantified at 24h by q-RT-PCR. Each point represents mean ± SD of triplicate cell culture.

As discussed above microbial IIRMIs are likely to be complex and activate multiple receptors. It has been postulated that synergy between two IIRMIs can occur when activating MyD88 dependent and MyD88-independent pathways, while competition for the same pathway or adaptor molecule might result in reduction of response [25–27]. Therefore we next explored whether the sensitivity to individual IIRMIs was modified by the presence of more than one IIRMIs using MM6 cells stimulated with a combination of endotoxin and FSL-1 or endotoxin and zymosan at different concentrations. As shown in Fig 7, the presence of multiple TLR ligands in the culture did not modify the sensitivity of MM6 cells to any one ligand as LLOD remained at 10pg endotoxin/mL, 100pg FSL-1/mL and 10ng zymosan/mL. Of note, in cells cultured in the presence of endotoxin and FSL-1–but not zymosan- we observed an increase in the expression of IL-6 mRNA as compared to endotoxin alone (Fig 7). This confirms that the presence of multiple ligands in a mixed culture can increase the magnitude of the cell response where as the LLOD for each individual impurity is unlikely to be modified.

## Discussion

Therapeutic proteins and peptides can contain multiple impurities, some of which are variants of the product, while others are derived from the cell substrate and the manufacturing process. While the former can provide additional epitopes, the latter, even when present at trace levels, have the potential to activate the PRR on local or systemic immune cells. Activation of PRR can lead to localized expression of cytokines and chemokines, increased antigen uptake, processing and presentation by APCs, and increased antibody secretion by B cells [28]. Thus, impurities in therapeutic products that can trigger PRRs can be considered as critical product attributes that can impact on the immunogenicity of product. Understanding the impurity profile of a product and their potential for increasing the risk of protein immunogenicity can help assess the safety of new products, established products that undergo manufacturing changes, and even biosimilars.

At present, routine testing to identify specific impurities is limited mostly to endotoxin, DNA and host cell proteins. [29]. The Rabbit Pyrogen Test (RPT) is one of the oldest methods for detecting impurities and is sensitive to wide range of pyrogens, however, it has important limitations. The RPT utilizes large number of laboratory animals and is unsuitable for testing products such as radiopharmaceuticals and chemotherapeutic agents, which would be toxic to the rabbits [30–31]. Lastly, the species differences in cellular distribution and sensitivity of PRRs can potentially hinder the interpretation of the results [32–33]. The Limulus Amoebocyte Lysate (LAL) assay is currently the method most frequently used to measure endotoxin as it is very sensitive and semi-quantitative; however immune-stimulating components from Gram-positive bacteria such as lipoproteins, peptidoglycan and lipoteichoic acids, flagellin or other pyrogens may pass the test without being detected. In addition, the presence of (1–3)- β-D-glucan in the test substance can interfere with the measurement of endotoxin [34–35]. Importantly, neither the RPT nor the LAL assays are designed to assess risk of product immunogenicity. It is only the more recent understanding of the innate immune system’s biology that dictates the need of assessing a broader spectrum of known and unknown IIRMIs in order to control or reduce the risk of unwanted immunogenicity by therapeutic proteins.

In the present study we propose the use of immune cell lines bearing PRRs to detect the presence of a broad range of IIRMIs in a sensitive manner. We present two different methods: 1) a panel of HEK-BLUE cell lines stably transfected with individual TLR and 2) the use of a combination of human and mouse macrophage cell lines that express multiple TLR among other PRRs (S1 Fig). Both systems allow for the recognition of TLR ligands with a sensitivity that is similar to human PBMC except for Poly I:C or MDP (Table 2). This suggests that the use of alternative cell lines may need to be considered to test products where the presence of TLR3 or NOD2 ligands is a concern. The first approach provides qualitative information on the type of IIRMIs but requires prior knowledge of the spectrum of possible impurities that could be present in the product. In contrast, professional antigen-presenting cell lines such as macrophages are armed with a broad array of PRRs and reasoned combinations results in sensitivity to a broad array of known or unknown impurities. It is possible to envision the use of both cell-based systems combined, where the macrophage lines are used to detect the presence of a broad range of impurities and the individually transfected cell lines may help identify the type of impurities present in the system.

While PBMC may be considered more representative of innate immune response *in vivo* than macrophage cell lines, the latter are readily available, easy to handle, not associated with adventitious agent as well as devoid of donor to donor variability. Indeed, the activation of NF-κB and increase in IL-6 and IL-8 by these cell lines, which are critical to the initiation of an immune response, suggests that the macrophage-based system is biologically relevant. Of note, the biological relevance of the cell based reporter system was confirmed by showing that concentrations of TLR agonists that induced NF-κB activation resulted in measurable increases in TNF-α in supernatants (S2 Fig). Further, our preliminary studies show that the concentrations of IIRMI that are identified by this assays is able to induce the activation of innate immune cells *in vivo* in the muscle of mice when injected intramuscular as detected by the induction of IL-1β mRNA (data not shown). This suggests that the levels of IIRMI that activate cells *in vitro* is biologically relevant and can induce local inflammation at the site of inoculation *in vivo* (Haile et al. manuscript in preparation).

Previous reports proposed the use of a single macrophage cell line based approach with Mono-Mac-6 (MM6) cells as an indicator of *in vitro* pyrogenicity [36–38]. According to our data, MM6 cells respond only to IIRMIs that signal through TLR1/2, TLR4 and TLR2/6 but not TLR3, TLR5, TLR7 or TLR9. Indeed, none of the cells we tested are considered to bear a sufficiently broad scope of PRRs to ensure that impurities were detected by a single cell line. Therefore, in this study, we proposed that multiple cells should be used and the selection should be partly driven by the product and its manufacturing process. Of note, in the studies that we performed, the presence of EPO or IFN-β did not impact the sensitivity or magnitude of cellular response to the impurities in the models tested; however, the sensitivity of the screening system for key impurities would need to be confirmed for each product as part of the assay validation exercise [39]. Confirming the sensitivity of the assay for the product will be particularly important for products that may modulate the activity of the cell lines directly.

In a previous study, we had shown that trace levels of IIRMIs could synergize to induce cytokine release and polyclonal B cell activation on murine splenocytes *in vitro* and increased antibody responses *in vivo* [5]. Here we show that the presence of multiple impurities did not modify the limit of detection of each individual impurity but it did enhance the magnitude of the biological read out; this is consistent with our previous observations in which IIRMI can synergistically induce a full blown immune response. Indeed, the observation that the presence of multiple IIRMI result in increased release of IL-6 and IL-8, which are evident in innate immune activation, suggests that the macrophage-based system is biologically relevant.

In summary, the use of multiple myeloid cell lines to screen products for the presence of innate immune response modifying impurities alone or in conjunction with cell lines bearing individual PRR appears to be a potentially useful tool for screening therapeutic proteins and peptides for the presence of impurities that may increase the risk of inducing an immune response. Over the last 15 years the recognition of the risk posed by immunogenicity to safety and efficacy of therapeutic proteins has resulted in the assessment of product immunogenicity for complex biologics during clinical trials or as a phase IV commitment becoming standard practice. However, the need for clinical studies to assess product immunogenicity as part of a comparability exercise following substantial manufacturing changes or for generic versions of complex products is based on a multifactorial assessment of risk that is highly predicated on the results of physic-chemical comparability studies. We propose that establishing the presence or absence of IIRMIs may contribute to this and other assessments of immunogenicity risk, such as for generic versions of complex products [40–41].

## Supporting Information

S1 Fig Expression of TLR by different macrophage lines. Total RNA was prepared from untreated RAW-BLUE, MM6 and differentiated THP1 cells and reverse transcribed. Expression of hTLR1-9 was measured by real-time PCR using Taqman primers/probes for each TLR. Data is expressed as the level of a particular TLR transcript normalized to 18S. Shown is mean delta Ct ± SE derived from 2 biological controls which run in duplicate.
(TIF)

S2 Fig RAW-BLUE cells detect trace levels of endotoxin in heparin lots. RAW-BLUE cells were cultured with 10 different lots of heparin (#1–10) in the presence or absence of 100pg/mL endotoxin for 24h. TNF-α levels were measured by ELISA in cell supernatant at 24 h. Each point represents mean ± SD of triplicate cell culture.
(TIFF)
