# Supplementary figures and images for: Detection of Innate Immune Response Modulating Impurities in Therapeutic Proteins
Source: PLoS One. 2015 Apr 22;10(4):e0125078. doi: 10.1371/journal.pone.0125078 (PMC4406594; doi:10.1371/journal.pone.0125078)

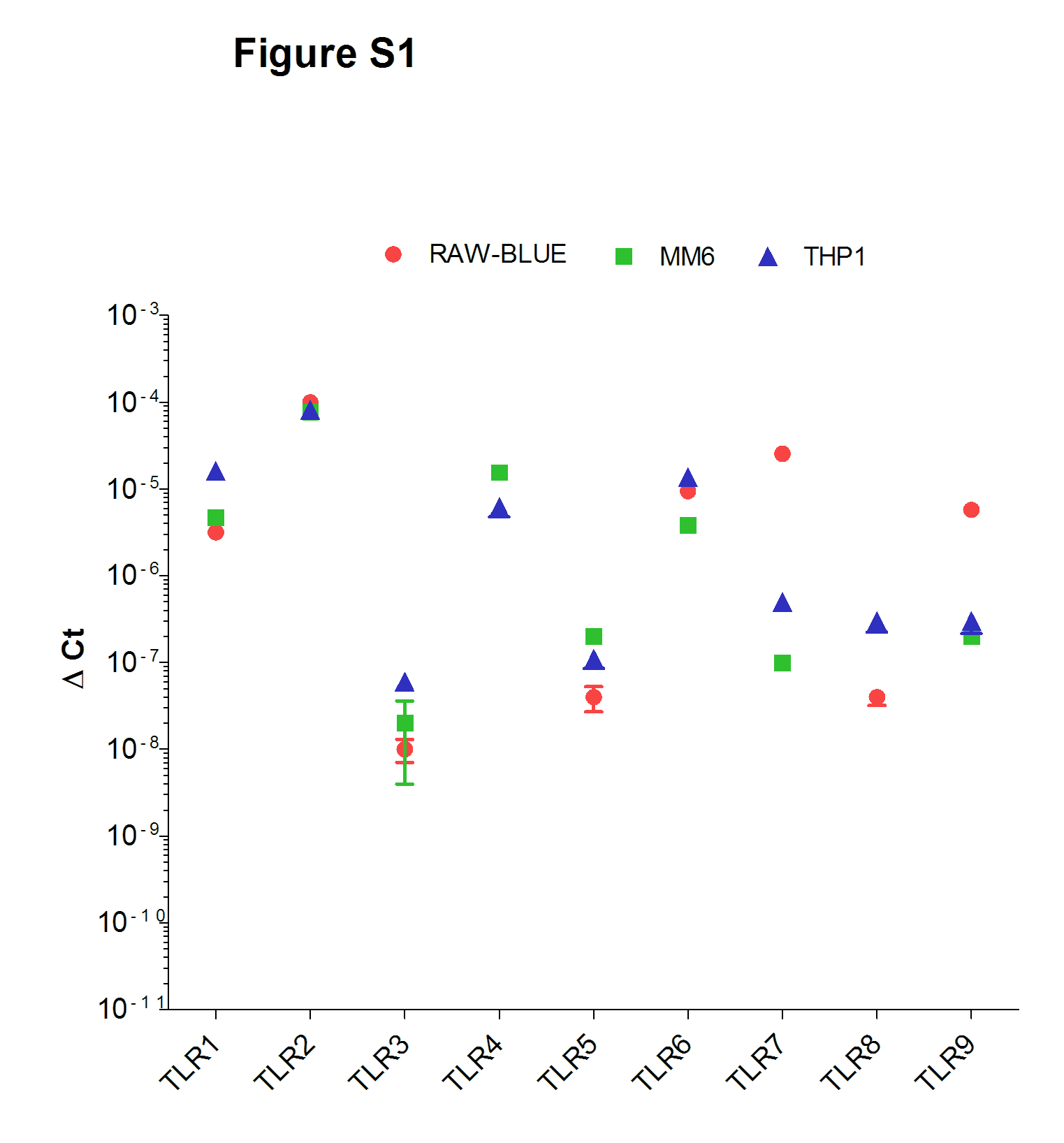

Supplement: S1 Fig — Total RNA was prepared from untreated RAW-BLUE, MM6 and differentiated THP1 cells and reverse transcribed. Expression of hTLR1-9 was measured by real-time PCR using Taqman primers/probes for each TLR. Data is expressed as the level of a particular TLR transcript normalized to 18S. Shown is mean delta Ct ± SE derived from 2 biological controls which run in duplicate. (TIF) [file pone.0125078.s001.tif]

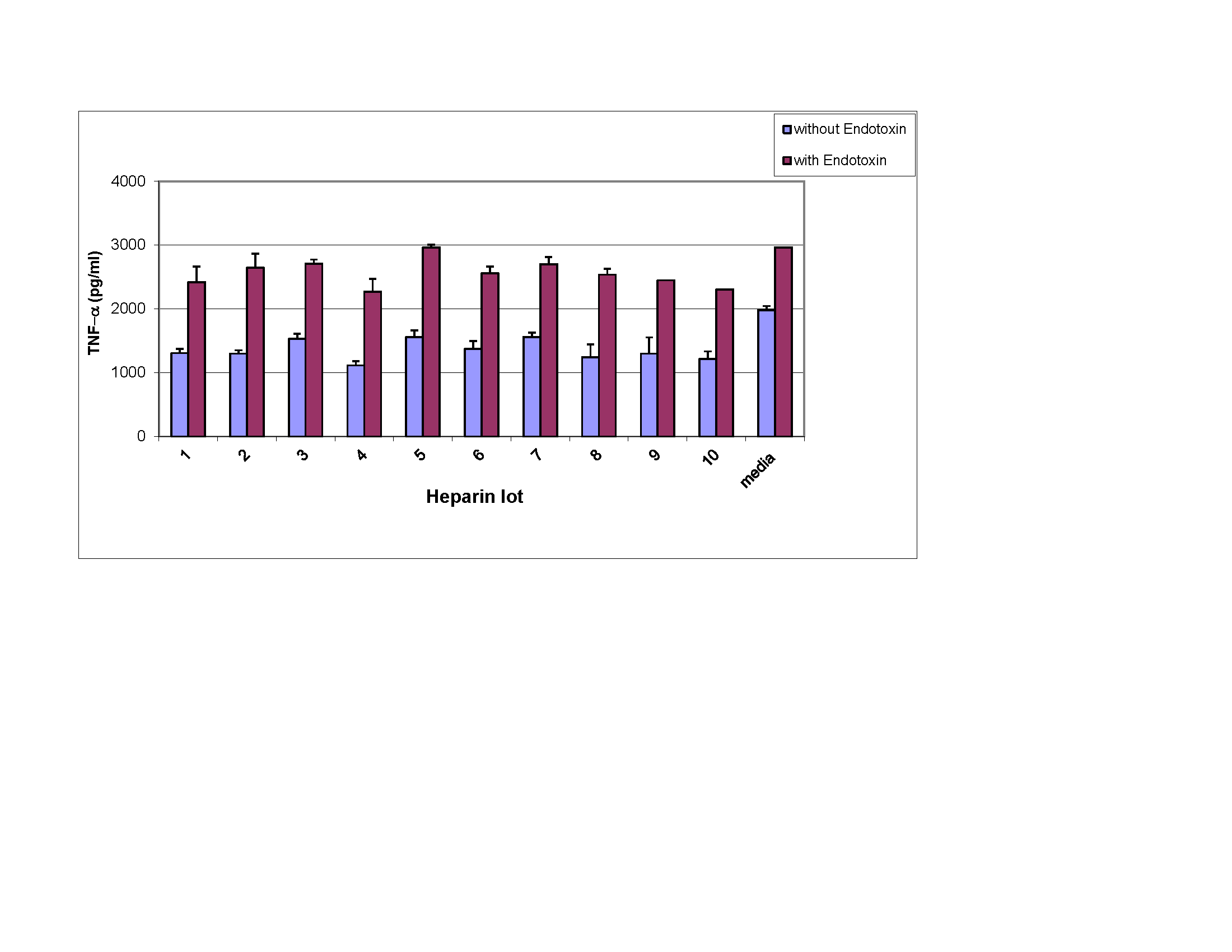

Supplement: S2 Fig — RAW-BLUE cells were cultured with 10 different lots of heparin (#1–10) in the presence or absence of 100pg/mL endotoxin for 24h. TNF-α levels were measured by ELISA in cell supernatant at 24 h. Each point represents mean ± SD of triplicate cell culture. (TIFF) [file pone.0125078.s002.tiff]
